# Supplementary material for: Free-space transfer of comb-rooted optical frequencies over an 18 km open-air link
Source: Nat Commun. 2019 Sep 30;10:4438. doi: 10.1038/s41467-019-12443-8 (PMC6769155; doi:10.1038/s41467-019-12443-8)
Supplement: Supplementary file 1 — Peer Review File [file 41467_2019_12443_MOESM1_ESM.pdf]

Editorial Note: This manuscript has been previously reviewed at another journal that is not operating a transparent peer review scheme. This document only contains reviewer comments and rebuttal letters for versions considered at Nature Communications

REVIEWERS' COMMENTS:

Reviewer #1 (Remarks to the Author):

The authors revised manuscript fully addresses and clarifies all questions. It is an impressive experiment and I definitely recommend publication.

Reviewer #2 (Remarks to the Author):

The authors sufficiently answered the questions of the referees.
I recommend publication as is.

<Response to referees' comments>.

[Authors' responses to Reviewer #1]

The authors revised manuscript fully addresses and clarifies all questions. It is an impressive experiment and I definitely recommend publication.

We appreciate the reviewer's positive comments. No revision is required.

[Authors' responses to Reviewer #2]

The authors sufficiently answered the questions of the referees. I recommend publication as is.

We appreciate the reviewer's positive comments. No revision is required.
